# *Ornithonyssus bacoti* Dermatitis Incorrectly Diagnosed as Delusional Parasitosis

**DOI:** 10.4269/ajtmh.23-0747

**Published:** 2024-03-19

**Authors:** Yangyang Ma, Wenting Hu

**Affiliations:** Department of Dermatology, Hangzhou Third People’s Hospital, Hangzhou, China

**Figure 1.**
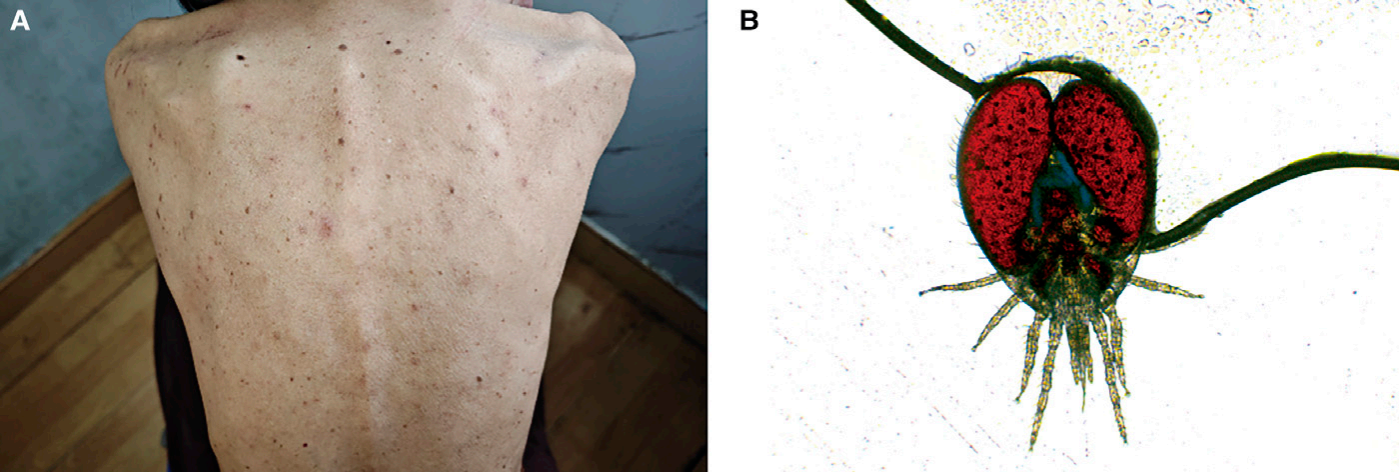
(**A**) Scattered erythema, papules, and scratch marks are visible on the patient's back. (**B**) Tropical rat mite under microscopy (×100).

A 70-year-old female patient was admitted with cutaneous pruritus and a reported parasite infestation over the past month. Previously diagnosed with delusional parasitosis at another hospital, dermoscopy and smears yielded no evidence of parasites or eggs. Physical examination revealed scattered erythematous papules on the trunk and limbs, accompanied by extensive scratch marks (Figure 1A). Despite prior negative findings, the patient insisted on the presence of parasite, claiming to catch more than 30 bugs daily. Upon closer inspection, bloodstains were found on her underwear, and shaking her clothes revealed the presence of parasites. Microscope examination confirmed *Ornithonyssus bacoti*, also referred to as the tropical rat mite (Figure 1B). The patient, who had a recent rat infestation at home, responded well to antiallergic treatment and environmental disinfection.

Tropical rat mite dermatitis, caused by *O. bacoti*, is a rat-origin parasitic disease. Unlike *Sarcoptes scabiei* or *Trombiculidae*, which inhabit human skin, *O. bacoti* is challenging to detect through routine dermatological examinations (scurf curettage and dermoscopy) due to its nonparasitic nature on human skin. Consequently, patients are often misdiagnosed with common dermatitis or delusional parasitosis when evidence of parasites is lacking.^1^ To improve detection rates, patients are advised to use tape to collect suspected insect for microscopic examination.^2^^,^^3^ We report here a rare case of *O. bacoti* infection, emphasizing the significance of bloodstains on underwear (resulting from crushed female mites after bloodsucking) and a history of rodent exposure are crucial diagnostic clues.
